# Functionalization of the BCL6 BTB domain into a noncovalent crystallization chaperone

**DOI:** 10.1107/S2052252520015754

**Published:** 2021-01-11

**Authors:** Thomas Zacharchenko, Stephanie Wright

**Affiliations:** a School of Biology and Astbury Centre for Structural Molecular Biology, University of Leeds, Woodhouse, Leeds LS2 9JT, United Kingdom

**Keywords:** protein crystallization, crystallization chaperone, BTB domain, porous crystal lattice

## Abstract

The production of diffraction-quality protein crystals is challenging and often requires bespoke, time-consuming and expensive strategies. A system has been developed in which the BCL6 BTB domain acts as a crystallization chaperone and promiscuous assembly block that may form the basis for affinity-capture crystallography. The protein of interest is expressed with a C-terminal tag that interacts with the BTB domain, and co-crystallization leads to its incorporation within a BTB-domain lattice. This strategy was used to solve the structure of the SH3 domain of human nebulin, a structure previously solved by NMR, at 1.56 Å resolution. This approach is simple and effective, requiring only routine protein complexation and crystallization screening, and should be applicable to a range of proteins.

## Abstract 2

The BCL6 BTB domain has been adapted to form a promiscuous assembly block as a basis for affinity-capture crystallography.

## 1. Introduction   

Protein crystallography remains the most accurate tool for the determination of high-resolution macromolecular structures. The main bottleneck in this technique is the production of well-ordered crystals that produce high-quality diffraction. Strategies to aid crystallization include the use of chaperones (Bukowska & Grütter, 2013 ▸; Ecsédi *et al.*, 2020 ▸), either in complex with the protein of interest or as a fusion partner, and the use of engineered lattices that accommodate the ‘guest’ protein. Lattices of defined dimensions should impose three-dimensional order on the captured guest molecule, serving both to aid crystallization and to act as a phasing tool for structure determination. This approach has successfully been applied to the structural determination of small molecules soaked into preformed three-dimensional metal–organic frameworks (Inokuma *et al.*, 2013 ▸); however, the application of this technology to protein structure determination is more challenging and requires lattices with much larger pores. A potential strategy is to incorporate the guest protein within an engineered protein lattice (Hamley, 2019 ▸), and a variety of approaches have been used to produce protein cages (Malay *et al.*, 2019 ▸), filaments (Shen *et al.*, 2018 ▸) and porous crystals of defined dimensions (Ward & Snow, 2020 ▸). Such assemblies have been achieved by the covalent modification of a target protein (Cattani *et al.*, 2015 ▸) or by the precise integration of symmetry elements (Padilla *et al.*, 2001 ▸; Sinclair *et al.*, 2011 ▸; Yeates *et al.*, 2016 ▸). Recently, the first example of incorporating a guest protein, ubiquitin, into a lattice was achieved by its genetic fusion to RIEN, a protein domain that naturally crystallizes into a porous honeycomb lattice with an internal diameter of ∼110 Å (Maita, 2018 ▸). The fusion protein crystallized in the same lattice as apo RIEN, and the structure of ubiquitin was solved using the phases calculated from RIEN; although impressive, the occupancy of ubiquitin was low, and crystal growth was only achieved after microseeding with apo RIEN. There is therefore a need for robust methods for achieving 100% occupancy of the guest protein and for the ready production of crystals through conventional sparse-matrix screening.

Strategies based on rigid predetermined scaffolds may be limited by the nature of the pores and by the availability of crystal contacts in the lattice, and there is therefore a need for more versatile approaches. Here, we report the functionalization of the BTB (bric à-brac, tramtrack and broad complex) domain from the transcriptional repressor BCL6 to serve as a promiscuous assembly block that may assist the crystallization of a guest protein. The 129-residue BCL6 BTB domain forms a symmetrical strand-exchanged homodimer that contains two lateral grooves that bind corepressors with high affinity (Ahmad *et al.*, 2003 ▸). By comparison of multiple crystal forms of the BCL6 BTB domain, we identified several regions of common crystallographic engagement, either with various co-crystallized peptides or between individual BTB molecules within the lattice. We showed that these can mediate the formation of a variety of highly porous lattices, and that a guest protein may be incorporated into a BTB-domain lattice by its genetic fusion to a corepressor peptide that binds to the lateral groove of the BTB domain with high affinity. We developed a modified BCL6 BTB domain as the first component of affinity-capture crystallography (ACC). In the second component, the protein of interest is expressed with a C-terminal tag that comprises the sequence of the natural BCL6-binding domain (BBD1) of the corepressor NCoR1. Co-purification of the two components enables crystallization of the protein of interest as a guest within a BTB-domain lattice. Using this strategy, we solved the structure of the SH3 domain of human nebulin, a structure previously determined by NMR (Politou *et al.*, 1998 ▸), to 1.56 Å resolution.

## 2. Materials and methods   

### 2.1. Peptides   

Peptides were synthesized by GL Biochem Shanghai Ltd. The sequences were ^1^GITTIKEMGRSIHEIPR^17^ (NCoR1^BBD1^) and ^1^RERIAAASSDLYLRPGS^17^ (NCoR1^BBD2^). The peptides were dissolved in appropriate buffers for NMR or crystallo­graphy and the pH was adjusted to experimental conditions prior to use.

### 2.2. Plasmids   

The human BCL6 BTB domain (encoding residues 6–129; UniProt accession P41182) was cloned into a modified pET-28a vector using ligation-independent cloning (In-Fusion, Clontech); the vector encoded an N-terminal hexahistidine-MBP tag. The point mutations C8Q, C67R and C84N were introduced by PCR using Phusion High-Fidelity DNA polymerase (Thermo Scientific); these mutations enhance protein solubility and have previously been used in crystallographic studies (Ahmad *et al.*, 2003 ▸). The NCoR1^BBD2^-BCL6^BTB^ and NCoR1^BBD2(link)^-BCL6^BTB^ chimeric proteins were made by fusing sequences encoding the residues SSDLYLRPG and SSDLYLRPGGG, respectively, onto the N-terminus of the BCL6 BTB domain using PCR. DNA sequences encoding the nebulin^SH3^-NCoR1^BBD1^ fusion protein were synthesized by GeneArt (Thermo Fisher Scientific) and encoded the nebulin SH3 domain (residues 6610–6669; UniProt accession P20929) fused to the NCoR1 BBD1 via a glycine linker (*GGG*GITTIKEMGRSIHEIPR); this was then cloned into the modified pET-28a vector as above. All constructs were verified by DNA sequencing (Genewiz).

### 2.3. Protein expression and purification   

Proteins were expressed in *Escherichia coli* BL21 (DE3) pLysS cells. The cultures were grown in 2YT medium at 310 K to an optical density (600 nm) of 0.6, cooled to 291 K and the recombinant proteins were induced by growth for a further 18 h in the presence of 500 µ*M* isopropyl β-d-1-thiogalactopyranoside. The cells were harvested by centrifugation at 11 000*g* and resuspended in 20 m*M* Na_2_HPO_4_, 500 m*M* NaCl, 40 m*M* imidazole, 3 m*M* β-mercaptoethanol pH 7.4. Bovine deoxyribonuclease 1 (Sigma) and Protease Inhibitor Cocktail VII (Merck) were added and the cells were lysed at 207 MPa using a cell disruptor (Constant Systems). The lysate was clarified by centrifugation at 39 000*g* for 45 min and the proteins were purified via immobilized metal-affinity chromatography using a 5 ml HisTrap HP column (GE Healthcare). The N-terminal His-MBP tag was removed by cleavage with HR3C protease, and the protein was further purified by size-exclusion chromatography on a Superdex 75 HiLoad 26/60 column (GE Healthcare). The NCoR1^BBD2(link)^-BCL6^BTB^/nebulin^SH3^-NCoR1^BBD1^ complex was obtained following size-exclusion chromatography of the mixed individual purified proteins, with nebulin^SH3^-NCoR1^BBD1^ in a 1.5:1 molar excess.

### 2.4. NMR spectroscopy   

NMR spectra were collected on a 750 MHz Bruker Avance spectrometer equipped with a cryoprobe. Data were collected at 298 K using 400 µ*M* protein in 20 m*M* Na_2_HPO_4_ pH 6.8, 300 m*M* NaCl, 3 m*M* β-mercaptoethanol.

### 2.5. X-ray crystallography   

All variations of the BCL6 protein (BCL6^BTB^, NCoR1^BBD2^-BCL6^BTB^ and NCoR1^BBD2(link)^-BCL6^BTB^) were concentrated to 300 µ*M* (monomer) in 20 m*M* HEPES pH 7.5, 300 m*M* NaCl, 3 m*M* β-mercaptoethanol, and all peptides were dissolved in the same buffer. For all crystallizations, standard sparse-matrix screening was carried out using 400 nl drops with a 1:1 protein:precipitant ratio at 298 K using a Mosquito Liquid Handling Robot (TTP Labtech). Initial hits for crystals of the BCL6^BTB^/NCoR1^BBD2^ complex were obtained in the conditions depicted in Supplementary Table S1.

For structure determination, crystals of the BCL6 BTB domain in complex with the high-affinity NCoR1^BBD1^ co­repressor peptide were obtained in 0.2 *M* sodium acetate trihydrate, 0.1 *M* bis-Tris propane 7.5, 20%(*w*/*v*) PEG 3350, with the NCoR1^BBD1^ peptide in a 4:1 molar excess. Crystals of the BCL6 BTB domain in complex with the low-affinity NCoR1^BBD2^ peptide were obtained in 13.4%(*v*/*v*) PEG 400, 0.335 *M* K_2_HPO_4_/NaH_2_PO_4_ pH 7.5, with the NCoR1^BBD2^ peptide in an 8:1 molar excess. Crystals of the NCoR1^BBD2^-BCL6^BTB^ chimera were obtained in 2.2 *M* NaCl, 10% MPD, 0.1 *M* imidazole pH 6.5, 0.1 *M* CaCl_2_. Crystals of the NCoR1^BBD2^-BCL6^BTB^ chimera in complex with the high-affinity NCoR1^BBD1^ peptide and of the NCoR1^BBD2(link)^-BCL6^BTB^ chimera in complex with the high-affinity NCoR1^BBD1^ peptide were obtained in 2 *M* NaCl, 5%(*w*/*v*) PEG 4000, 0.1 *M* Tris–HCl pH 8.5 and in 1.34 *M* ammonium sulfate, 0.67%(*v*/*v*) MPD, 0.1 *M* HEPES/sodium hydroxide pH 7.5, respectively, each with the NCoR1^BBD1^ peptide in a 4:1 molar excess. The NCoR1^BBD2(link)^-BCL6^BTB^ chimera in complex with nebulin^SH3^-NCoR1^BBD1^ was purified by size-exclusion chromatography and concentrated to 7 mg ml^−1^ (determined using an assumption of 1:1 stoichiometry and combined extinction coefficients). Crystals were obtained in 0.66 *M* ammonium sulfate, 3.3%(*v*/*v*) glycerol, 0.05 *M* magnesium sulfate, 0.1 *M* imidazole–HCl pH 6.5. Prior to data collection, all crystals were vitrified in mother liquor containing 25% ethylene glycol and were rapidly cooled using liquid nitrogen.

X-ray data from individual crystals were collected to a range of resolutions from 1.39 to 3.25 Å, with data reduction and integration performed using either *AIMLESS* (Evans & Murshudov, 2013 ▸), *SCALA* (Evans, 2006 ▸) or the *xia*2 -3d pipeline (Winter, 2010 ▸). The structures were readily solved by molecular replacement in *Phaser* (McCoy *et al.*, 2007 ▸) using PDB entry 1r28 as the template model (Ahmad *et al.*, 2003 ▸) and refined using *Phenix* 1.17 (Liebschner *et al.*, 2019 ▸). The crystal structure of the NCoR1^BBD2(link)^-BCL6^BTB^ chimera in complex with the NCoR1^BBD1^ corepressor peptide showed signs of pseudotranslational NCS. Examination of the data suggested that the crystal may belong to space group *P*4_1_2_1_2, with a Patterson off-origin peak of 83.7% at fractional coordinates 0.5, 0.5, 0.5 and a distance of 151.4 Å from the origin. Analysis of the acentric reflections also showed that 〈*I* ^2^〉/〈*I*〉^2^ = 3.035, 〈*F*〉^2^/〈*F* ^2^〉 = 0.665 and 〈|*E* ^2^ − 1|〉 = 0.990; for the centric reflections 〈*I* ^2^〉/〈*I*〉^2^ = 3.904, 〈*F*〉^2^/〈*F* ^2^〉 = 0.585 and 〈|*E* ^2^ − 1|〉 = 1.153. In space group *P*4_1_2_1_2, six BTB monomers were present in the asymmetric unit, and were placed using a combination of *Phaser* and *MOLREP* (Vagin & Teplyakov, 2010 ▸). Matthews water coefficients were calculated using the theoretical molecular weights of constructs using *CCP*4 (Winn *et al.*, 2011 ▸).

All models were built using *Coot* (Emsley *et al.*, 2010 ▸), with iterative rounds of refinement in *Phenix* 1.17. Data-reduction and refinement statistics are shown in Table 1 ▸.

The structures were deposited in the PDB with the following accession codes: BCL6^BTB^/NCoR1^BBD1^, 6xyx; BCL6^BTB^/NCoR1^BBD2^, 6xzz; NCoR1^BBD2^-BCL6^BTB^, 6xwf; NCoR1^BBD2^-BCL6^BTB^/NCoR1^BBD1^, 6xxs; NCoR1^BBD2(link)^-BCL6^BTB^/NCoR1^BBD1^, 6zbu; NCoR1^BBD2(link)^-BCL6^BTB^/nebulin^SH3^-NCoR1^BBD1^, 6y17.

## 3. Results and discussion   

### 3.1. Low-affinity interactions of the NCoR1 corepressor with the BCL6 BTB domain   

**Figure 1:**
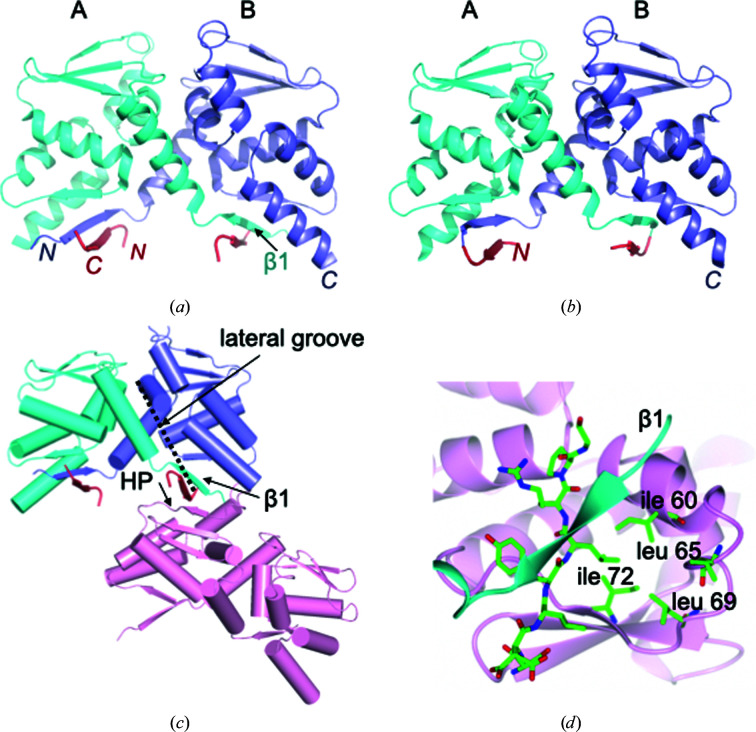
Crystal structures of the BCL6 BTB domain in complex with NCoR1 BBD2 sequences. (*a*) The BCL6 BTB-domain dimer in complex with the low-affinity NCoR1^BBD2^ peptide. (*b*) The NCoR1^BBD2^-BCL6^BTB^ chimeric protein. (*c*) The NCoR1^BBD2^ peptide interacts with two BTB-domain dimers within the crystal lattice. (*d*) Interfacing residues between the NCoR1^BBD2^ peptide and the BCL6 BTB domain. The cartoon representations in (*a*) and (*b*) depict chains of the domain-swapped BTB-domain dimer in blue and cyan and NCoR1^BBD2^ residues in red. The NCoR1^BBD2^ sequences interact with both the β1 strand and hydrophobic patch (HP) region of the BTB domain, thereby tethering two BTB dimers together. The position of the lateral groove of the BTB domain is indicated.

As a starting point for this work, in a parallel line of enquiry we unexpectedly observed a low-affinity interaction between the BCL6 BTB domain and a 17-residue peptide derived from the NCoR1 corepressor by 2D NMR (Supplementary Fig. S1). We refer to this NCoR1 sequence as BBD2; it is distinct from the NCoR1 sequence BBD1 which binds to the lateral groove of the BTB domain with high affinity (Ahmad *et al.*, 2003 ▸). The low-affinity NCoR1^BBD2^ peptide, ^1^RERIAAASSDLYLRPGS^17^, significantly improved the crystallization of the BCL6 BTB domain, giving initial hits in 21 conditions of a sparse-matrix screen (Supplementary Table S1). The structure of the BCL6^BTB^/NCoR1^BBD2^ complex was solved to 1.39 Å resolution [Fig. 1 ▸(*a*) and Table 1 ▸]; only the SDLYLRPG sequence of the peptide was resolved. We sought to determine whether this sequence could promote crystallization of the BCL6 BTB domain when fused directly to it, and made a chimeric BTB protein in which SSDLYLRPG was covalently linked to its N-terminus (NCoR1^BBD2^-BCL6^BTB^). The SSDLYLRPG sequence similarly enhanced both crystallization and diffraction quality in this context, and we solved the structure of the NCoR1^BBD2^-BCL6^BTB^ chimera to 1.6 Å resolution [Fig. 1 ▸(*b*) and Table 1 ▸]. Crystallization of both the BCL6^BTB^/NCoR1^BBD2^ complex and the NCoR1^BBD2^-BCL6^BTB^ chimera was favoured in conditions of high ionic strength, and the crystals had similar cell dimensions and the same packing in space group *P*6_1_22.

In the crystals of the BCL6^BTB^/NCoR1^BBD2^ complex and of the NCoR1^BBD2^-BCL6^BTB^ chimera, the NCoR1^BBD2^ sequence simultaneously interacts with two regions of the BTB domain: the first is the β1 strand located in the lower part of the lateral groove of the BTB domain and the second is a region we refer to as the hydrophobic patch (HP) located on the surface of the BTB domain between residues 60 and 72. A peptide molecule thereby tethers two BTB dimers together in the lattice [Figs. 1 ▸(*c*) and 1 ▸(*d*)].

### 3.2. Crystallization of the BCL6 BTB domain into a highly porous lattice   

**Figure 2:**
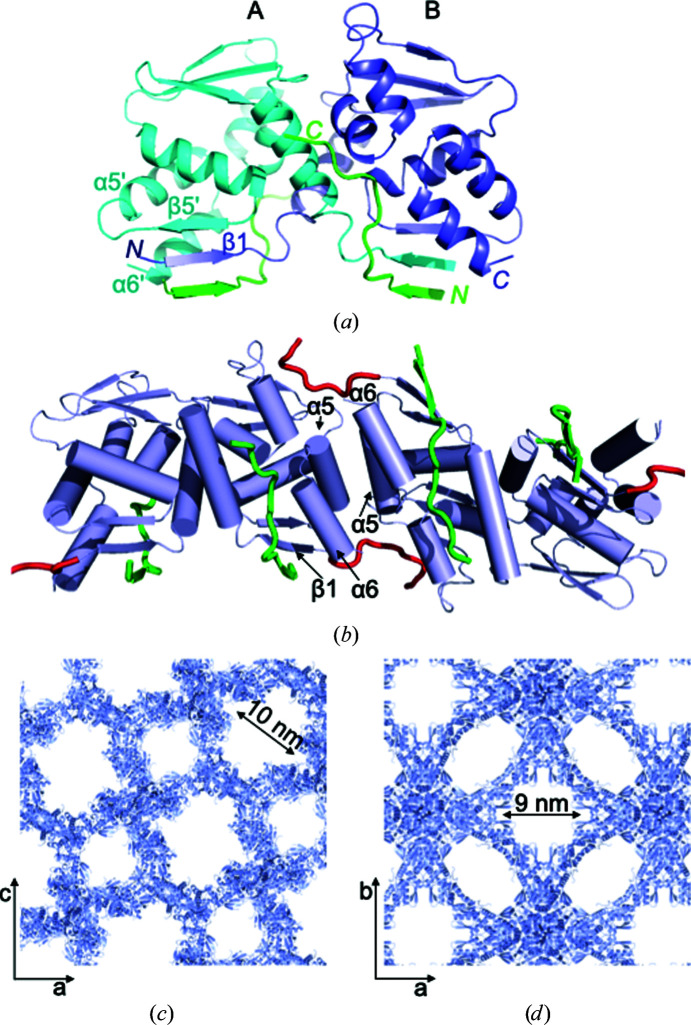
Crystallization of the BCL6 BTB domain into a highly porous lattice. (*a*) Structure of the BCL6 BTB domain in complex with the high-affinity NCoR1^BBD1^ peptide. The β1 strand of the *B* chain is indicated; β5, α5 and α6 of the *A* chain are indicated with a prime. BCL6 chains are depicted in blue and cyan and NCoR1^BBD1^ in green. (*b*) The interface between BTB-domain dimers in crystals of the NCoR1^BBD2^-BCL6^BTB^/NCoR1^BBD1^ complex; BCL6 chains are depicted in blue, NCoR1^BBD1^ in green and NCoR1^BBD2^ in red. (*c*) Crystal packing of NCoR1^BBD2^-BCL6^BTB^/NCoR1^BBD1^, showing a projection across the *ac* face. (*d*) Crystal packing of NCoR1^BBD2(link)^-BCL6^BTB^/NCoR1^BBD1^, showing a projection across the *ab* face.

We next sought to adapt the BTB domain to form an assembly that might act as a crystallographic scaffold for hosting a guest protein of interest. By solving the crystal structure, we showed that the classic high-affinity NCoR1 BTB-binding sequence, NCoR1^BBD1^ (Ahmad *et al.*, 2003 ▸), interacts with the lateral groove of the BTB domain identically to the highly homologous corepressor SMRT [Fig. 2 ▸(*a*) and Table 1 ▸]; the region of interaction includes the BTB-domain β1 strand that was bound by NCoR1^BBD2^ in our previous structures. We reasoned that the high-affinity NCoR1^BBD1^ peptide would displace the NCoR1^BBD2^ residues from the BTB-domain β1 strand of the NCoR1^BBD2^-BCL6^BTB^ chimera, whilst still leaving the NCoR1^BBD2^ sequence free to potentially interact with the HP of an adjacent BTB domain; this might result in the effective polymerization of the BTB homodimer (Nauli *et al.*, 2007 ▸). We crystallized the NCoR1^BBD2^-BCL6^BTB^ chimera in the presence of the high-affinity NCoR1^BBD1^ peptide and solved the structure of the NCoR1^BBD2^-BCL6^BTB^/NCoR1^BBD1^ complex to 3.25 Å resolution [Fig. 2 ▸(*b*), Supplementary Fig. S3(*a*) and Table 1 ▸]. The high-affinity NCoR1^BBD1^ peptide interacted with the lateral groove of the BTB domain, and the N-terminal NCoR1^BBD2^ extension of the NCoR1^BBD2^-BCL6^BTB^ chimera was displaced from the β1 strand as expected. The NCoR1^BBD2^ sequence interacted with the HP region of a neighbouring BTB dimer, and the BTB domains were arranged in distinct filaments that were formed by the back-to-back association of four BTB dimers [Supplementary Figs. S3(*a*) and S4]. These interactions involved the C-terminal α5–α6 helices of the BTB domain; similar associations of this region have been observed in another crystal structure of the BCL6 BTB domain [BCL6^BTB^/BCOR^BBD^; PDB entry 3bim; Supplementary Fig. S3(*b*)] (Ghetu *et al.*, 2008 ▸). The assembly of BTB dimers into filaments therefore involved two types of association: firstly the end-to-end stacking mediated by α5–α6 and secondly the association of the NCoR1^BBD2^ appendage with the HP region of an adjacent BTB molecule. This latter type of association between adjacent molecules has been described as ‘runaway domain coupling’ when occurring in solution *in vivo* (McPartland *et al.*, 2018 ▸); the strategy of combining runaway domain coupling with domain stacking leads to a stronger association between units and has been described in the formation of filaments from globular proteins. The association of the NCoR1^BBD2^ appendage with the BTB-domain HP region increases the total buried surface area of the interface between adjacent BTB dimers from 1005.8 Å^2^ (PDB entry 3bim) to 2215.2 Å^2^ (NCoR1^BBD2^-BCL6^BTB^/NCoR1^BBD1^).

The crystals of the NCoR1^BBD2^-BCL6^BTB^/NCoR1^BBD1^ complex were highly porous, with a solvent content of 82.30% (*V*_M_ = 6.95 Å^3^ Da^−1^). The N-termini of the NCoR1^BBD1^ peptides were exposed in solvent channels of ∼100 Å in diameter as estimated from projections across the crystal lattice in *CCP*4*mg* [Fig. 2 ▸(*c*)]. *MAP_CHANNELS* analysis revealed solvent channels of diameter ∼50 Å (Supplementary Fig. S2), and we therefore reasoned that we could adapt this system for the accommodation of a guest protein that is expressed as a genetic fusion to the NCoR1^BBD1^ sequence.

We next attempted to increase the affinity between the back-to-back associations of BTB domains in the lattice, with the intention of creating indefinitely long filaments that would form a scaffold for the recruitment of guest proteins. Such an extension might also allow flexibility and versatility in lattice assembly. We therefore introduced two additional glycine residues between the NCoR1^BBD2^ sequence and the BTB domain within the NCoR1^BBD2^-BCL6^BTB^ chimera, with the rationale being to extend the interface with the HP region. The modified chimeric protein, NCoR1^BBD2(link)^-BCL6^BTB^, was crystallized in complex with the high-affinity NCoR1^BBD1^ peptide. We solved the structure of the NCoR1^BBD2(link)^-BCL6^BTB^/NCoR1^BBD1^ complex at 2.46 Å resolution; these crystals had a similar solvent content (76.83%; *V*_M_ = 5.31 Å^3^ Da^−1^) to the previous complex and formed a similar porous lattice [Fig. 2 ▸(*d*)]. As expected, the interface between the NCoR1^BBD2^ sequence and the HP region of the BTB domain was extended in this structure [Supplementary Fig. S3(*d*)] and the BTB domains were arranged in indefinitely long filaments throughout the crystal lattice. The N-termini of the NCoR1^BBD1^ peptides were exposed in the solvent channels as before. Thus, upon crystallization in the presence of the high-affinity NCoR1^BBD1^ peptide, the two chimeric BTB domains each assembled into filaments that formed open lattices. This system might therefore be used to accommodate a guest protein that is recruited to the BTB domain via its genetic fusion to the NCoR1^BBD1^ sequence (Supplementary Fig. S5).

### 3.3. Using the BCL6 BTB domain as a crystallization chaperone   

**Figure 3:**
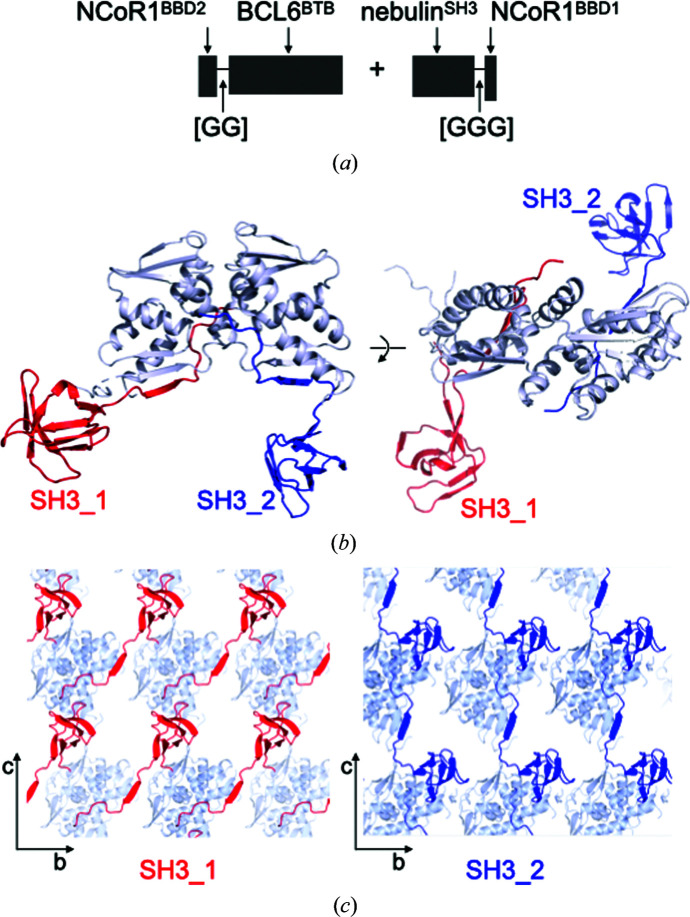
The BCL6 BTB domain as a crystallization chaperone. (*a*) Schematic representation of fusion proteins. (*b*) Structure of the NCoR1^BBD2(link)^-BCL6^BTB^/nebulin^SH3^-NCoR1^BBD1^ complex. BCL6 BTB dimers are shown in white/blue and NCoR1^BBD1^-nebulin^SH3^ sequences in red and dark blue. (*c*) Crystal packing of the two SH3 domains.

As proof of principle, we chose to crystallize the SH3 domain of human nebulin by incorporating it as the guest protein within a BCL6 BTB-domain lattice via its genetic fusion to the NCoR1^BBD1^ sequence; the nebulin SH3 domain structure has previously been solved by NMR (Politou *et al.*, 1998 ▸), thereby providing solution-state information to enable the determination of potential artefactual conformations induced by the scaffold, and it has a largest dimension of ∼28 Å. We expressed the nebulin SH3 domain as a fusion protein in which the NCoR1^BBD1^ sequence was tethered to its C-terminus; a linker of three glycine residues was inserted between the two sequences to reduce potential steric clashes of the SH3 domain with the scaffold [Fig. 3 ▸(*a*)]. The NCoR1^BBD2(link)^-BCL6^BTB^ and nebulin^SH3^-NCoR1^BBD1^ proteins were expressed and co-purified as a complex using size-exclusion chromatography [Supplementary Fig. S6(*a*)], and we obtained multiple hits using sparse-matrix crystallization screening. Using diffraction data from large rod-shaped crystals that grew within approximately one week [Supplementary Fig. S6(*b*)], we performed molecular replacement using the BCL6 BTB-domain homodimer (PDB entry 1r28; Ahmad *et al.*, 2003 ▸) as a search model, thereby placing one BTB dimer within the unit cell. Electron density corresponding to two SH3 domains was visible in the *F*_o_ − *F*_c_ difference map [Supplementary Fig. S6(*c*)], allowing the complete *de novo* construction of both the SH3 domains and the NCoR1^BBD1^ sequence [Fig. 3 ▸(*b*), Supplementary Fig. S6(*e*) and Table 1 ▸]. The structure of the nebulin SH3 domain was resolved to 1.56 Å resolution. Comparison with the NMR structure (PDB entry 1neb) reveals a backbone r.m.s.d. of 1.12 Å^2^ [Supplementary Fig. S6(*d*)]. Each BTB dimer recruited two nebulin^SH3^-NCoR1^BBD1^ molecules, and the NCoR1^BBD1^ moieties occupied the lateral groove and interacted with the BTB β1 strands as expected [Fig. 3 ▸(*b*)]. Unexpectedly, the BTB dimers did not assemble into filaments; instead, the positioning of the SH3 domains within the lattice precluded the back-to-back associations between BTB dimers, with one of the SH3 domains interacting with the BTB domain HP [Fig. 3 ▸(*c*)]. The versatility of the BTB-domain crystal contacts thus allowed the incorporation of the guest nebulin protein even though it drove a change in lattice organization.

## 4. Conclusions   

We have shown that the surface of the BCL6 BTB domain contains versatile interaction interfaces with the potential to mediate diverse lattices in a range of crystallization conditions. The hydrophobic patch of the BTB domain provided a site for interaction either with the NCoR1^BBD2^ appendage or with a co-crystallized SH3 domain that was recruited via genetic fusion to a natural NCoR1 corepressor BBD1 sequence; from this perspective, the BTB domain served as a chaperone for the SH3 domain and provided an interaction site to facilitate the 3D ordering of the crystal. Although unexpected, the propensity of the HP to interact with a variety of hydrophobic sequences confers versatility upon the organization of BTB-domain lattices, thereby offering advantages over strategies that involve the recruitment of guest proteins into precisely defined scaffolds. For example, the crystallization of ubiquitin by its genetic fusion to RIEN required microseeding with apo RIEN (Maita, 2018 ▸), and the low occupancy presumably reflected the difficulty in incorporating the guest ubiquitin protein into the fixed RIEN scaffold.

We propose that the modified BCL6 BTB domain represents a promiscuous assembly block that may form the basis for affinity-capture crystallography, whereby a protein of interest is captured into a BTB lattice via its genetic fusion to the BTB-binding corepressor BBD1 sequence. We anticipate that the versatility of BTB lattices will confer advantages over systems that use precisely defined rigid scaffolds, where crystallization may be prevented if the guest protein disrupts self-assembly. We therefore consider it likely that the BTB scaffold will be particularly beneficial for the co-crystallization of larger proteins. Future work will optimize this system for the crystallization of a variety of guest proteins.

## Supplementary Material

PDB reference: BCL6^BTB^/NCoR1^BBD1^, 6xyx

PDB reference: BCL6^BTB^/NCoR1^BBD2^, 6xzz

PDB reference: NCoR1^BBD2^-BCL6^BTB^, 6xwf

PDB reference: NCoR1^BBD2^-BCL6^BTB^/NCoR1^BBD1^, 6xxs

PDB reference: NCoR1^BBD2(link)^-BCL6^BTB^/NCoR1^BBD1^, 6zbu

PDB reference: NCoR1^BBD2(link)^-BCL6^BTB^/nebulin^SH3^-NCoR1^BBD1^, 6y17

Supplementary Tables and Figures. DOI: 10.1107/S2052252520015754/lz5043sup1.pdf

## Figures and Tables

**Table 1:** Crystallographic statistics Values in parentheses are for the highest resolution shell.

	BCL6^BTB^/NCoR1^BBD2^	NCoR1^BBD2^-BCL6^BTB^	BCL6^BTB^/NCoR1^BBD1^	NCoR1^BBD2^-BCL6^BTB^/NCoR1^BBD1^	NCoR1^BBD2(link)^-BCL6^BTB^/NCoR1^BBD1^	NCoR1^BBD2(link)^-BCL6^BTB^/nebulin^SH3^-NCoR1^BBD1^
PDB code	6xzz	6xwf	6xyx	6xxs	6zbu	6y17
Diffraction data						
Beamline	I04, Diamond	I24, Diamond	I04, Diamond	I24, Diamond	ID30A-1, ESRF	I24, Diamond
Wavelength (Å)	0.9795	0.9686	0.9795	0.9686	0.966	0.9688
Resolution (Å)	54.22–1.39 (1.41–1.39)	59.70–1.60 (1.69–1.60)	21.63–1.44 (1.52–1.44)	93.05–3.25 (3.43–3.25)	98.32–2.46 (2.52–2.46)	46.88–1.56 (1.60–1.56)
Space group	*P*6_1_22	*P*6_1_22	*P*2_1_2_1_2_1_	*P*6_5_22	*P*4_1_2_1_2	*P*1
Unit-cell parameters						
*a* (Å)	67.01	68.94	34.66	165.38	198.57	39.46
*b* (Å)	67.01	68.94	64.52	165.38	198.57	47.30
*c* (Å)	152.01	166.91	137.75	244.80	113.07	59.78
α (°)	90	90	90	90	90	95.87
β (°)	90	90	90	90	90	95.60
γ (°)	120	120	90	120	90	94.20
Unique reflections	41626 (2036)	31944 (4539)	56791 (8133)	31807 (4532)	82349 (6006)	58832 (4261)
Completeness (%)	100 (99.7)	100 (100)	99.7 (99.8)	100 (100)	99.9 (100)	96.9 (95.1)
Multiplicity	18.3 (18.2)	33.6 (34.6)	3.8 (3.7)	8.9 (9.1)	5.8 (5.9)	3.4 (3.0)
CC_1/2_	0.999 (0.524)	0.999 (0.465)	0.998 (0.606)	0.958 (0.154)	0.997 (0.345)	0.989 (0.383)
〈*I*/σ(*I*)〉	13.2 (1.3)	14.3 (1.8)	9.1 (1.4)	5.5 (1.1)	6.9 (1.3)	5.5 (1.5)
*R*_merge_(*I*)	0.157 (5.993)	0.191 (2.983)	0.068 (0.894)	0.479 (2.318)	0.150 (1.315)	0.118 (1.372)
*R*_p.i.m._	0.038 (1.439)	0.033 (0.511)	0.034 (0.530)	0.170 (0.818)	0.067 (0.593)	0.075 (0.955)
Refinement						
*R*_work_ (%)	18.47	19.01	16.70	19.66	22.30	17.66
*R*_free_ (%)	20.51	21.80	19.77	22.46	25.90	20.08
No. of atoms						
Total	1257	1197	2506	4693	7421	3654
Macromolecule	1120	1045	2256	4693	7137	3291
Solvent	137	152	250	0	120	363
R.m.s.d., bonds (Å)	0.005	0.005	0.005	0.003	0.010	0.008
R.m.s.d., angles (°)	0.808	0.709	0.691	0.575	1.158	0.945
Whole-chain *B* factor (Å^2^)						
Chain *A*	19.74	29.83	28.46	69.60	59.80	23.65
Chain *B*	25.92		31.16	72.03	55.93	24.81
Chain *C*			36.44	70.42	63.31	34.85
Chain *D*			30.80	71.33	69.16	26.55
Chain *E*				80.70	63.36	
Chain *F*				87.51	79.23	
Chain *G*				94.44	77.75	
Chain *H*				104.32	93.83	
Chain *I*					65.24	
Chain *J*					86.77	
Chain *K*					79.10	
Chain *L*					101.14	
Ramachandran						
Favoured (%)	98.44	99.21	98.47	95.20	96.12	98.77
Allowed (%)	1.56	0.79	1.53	4.62	3.17	1.23
