# Supplementary material for: Functionalization of the BCL6 BTB domain into a noncovalent crystallization chaperone
Source: IUCrJ. 2021 Jan 11;8(Pt 2):154–60. doi: 10.1107/S2052252520015754 (PMC7924223; doi:10.1107/S2052252520015754)
Supplement: Supplementary file 1 [file m-08-00154-sup1.pdf]

IUCrJ

Volume 8 (2021)

Supporting information for article:

Functionalization of the BCL6 BTB domain into a noncovalent crystallization chaperone

Thomas Zacharchenko and Stephanie Wright

Table S1 BCL6^{BTB} + NCoR1^{BBD2} crystallization conditions

Sparse matrix screening conditions that produced initial hits for crystals of BCL6^{BTB} in complex with the NCoR1^{BBD2} peptide.

Condition
2500 mM sodium chloride, 100 mM potassium phosphate monobasic/sodium phosphate dibasic pH 6.2
2500 mM sodium chloride, 100 mM Tris base/hydrochloric acid pH 7.0, 200 mM magnesium chloride
1260 mM ammonium sulfate, 100 mM CHES/sodium hydroxide pH 9.5, 200 mM sodium chloride
2000 mM ammonium sulfate, 100 mM CAPS/sodium hydroxide pH 10.5, 200 mM lithium sulfate
35% (v/v) MPD, 100 mM Tris base/hydrochloric acid pH 7.0, 200 mM sodium chloride
2000 mM ammonium sulfate, 100 mM CAPS/sodium hydroxide pH 10.5, 200 mM lithium sulfate
20% (w/v) PEG 8000, 100 mM Tris base/hydrochloric acid pH 8.5, 200 mM magnesium chloride
2000 mM ammonium sulfate, 100 mM sodium cacodylate/hydrochloric acid pH 6.5, 200 mM sodium chloride
0.66 M ammonium sulfate, 0.66%(v/v) PEG 400, 0.1 M sodium acetate/acetic acid pH 5.5
1.34 M ammonium sulfate, 0.67%(v/v) MPD, 0.1M HEPES/sodium hydroxide pH 7.5
2 M ammonium sulfate 5%(v/v), PEG 400, 0.05 M magnesium sulfate, 0.1M Tris base/hydrochloric acid pH 8.5
1.34 M ammonium sulfate, 3.35%(v/v) PEG 400, 0.05 M magnesium sulfate, 0.1 M Tris base/hydrochloric acid pH 8.5
2.01 M sodium chloride, 3.35%(v/v) MPD, 0.1 M calcium chloride, 0.1 M imidazole/hydrochloric acid pH 6.5
2.68 M Sodium chloride, 3.35%(v/v) isopropanol, 0.1M HEPES/sodium hydroxide pH 7.5
3.35%(v/v) isopropanol, 1.675 M potassium phosphate dibasic/sodium phosphate monobasic pH 5.5
2 M lithium sulfate, 2%(v/v) PEG 400, 0.1 M Tris base/hydrochloric acid pH 8.5
0.495 M ammonium sulfate, 3.96%(v/v) isopropanol, 0.1 M imidazole/hydrochloric acid pH 6.5
9.9%(w/v) PEG 1500, 0.99%(v/v) MPD, 0.2 M magnesium sulfate, 0.1 M sodium acetate/acetic acid pH 5.5
13.4%(w/v) PEG 8000, 13.4%(v/v) PEG 400, 0.1 M magnesium chloride, 0.1 M Tris base/hydrochloric acid pH 8.5
0.67%(v/v) PEG 4000, 0.67 M ammonium citrate/citric acid pH 5.5
0.825 M sodium chloride, 3.96%(w/v) PEG 1500, 0.495%(v/v) MPD, 0.1 M sodium acetate/acetic acid pH 5.5

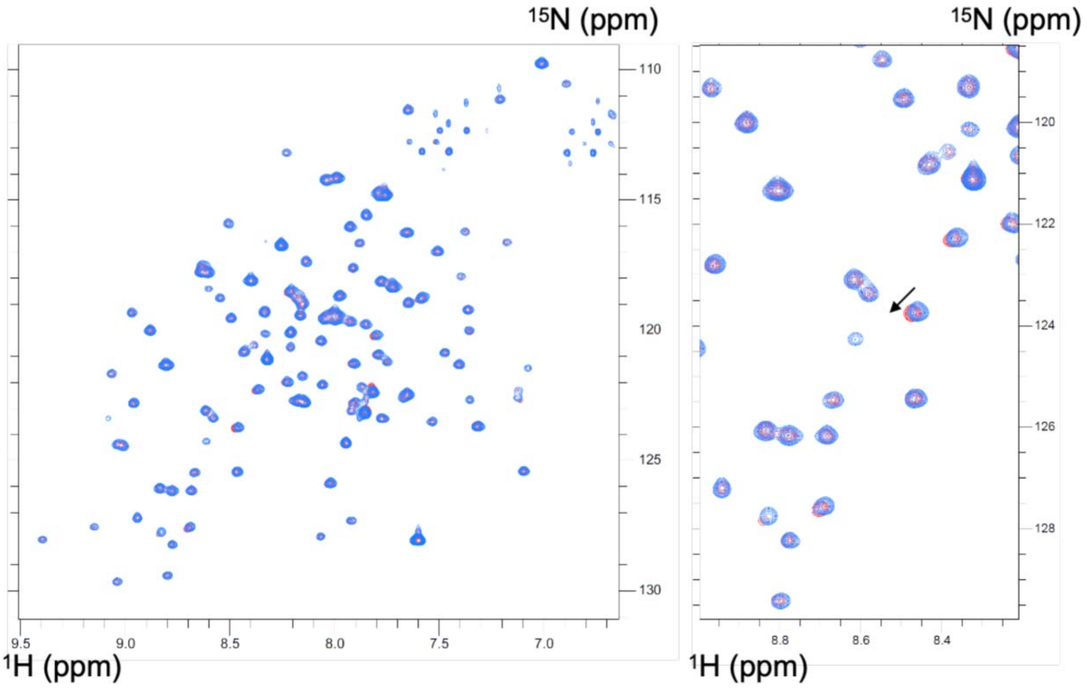

Figure S1 The NCoR1^{BBD2} peptide binds the BCL6 BTB domain with low affinity, ^{1}H-^{15}N TROSY HSQC spectra of BCL6^{BTB} (blue) titrated with an 8-fold excess of NCoR1^{BBD2} peptide (red).

(a)

(b)

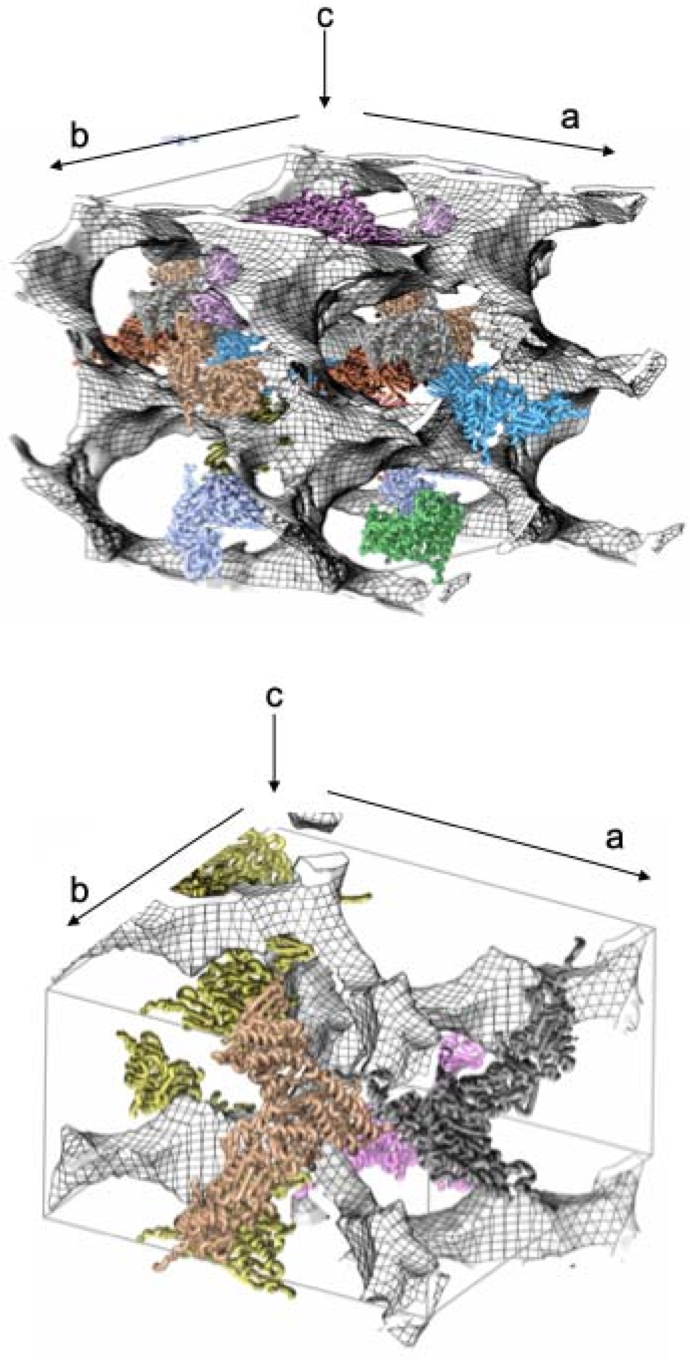

Figure S2 *MAP_CHANNELS* analysis of crystal structures. MAP_CHANNELS analysis of (a) 6XXS and (b) 6ZBU, showing the unit cell and a map contour radius of 25 Å.

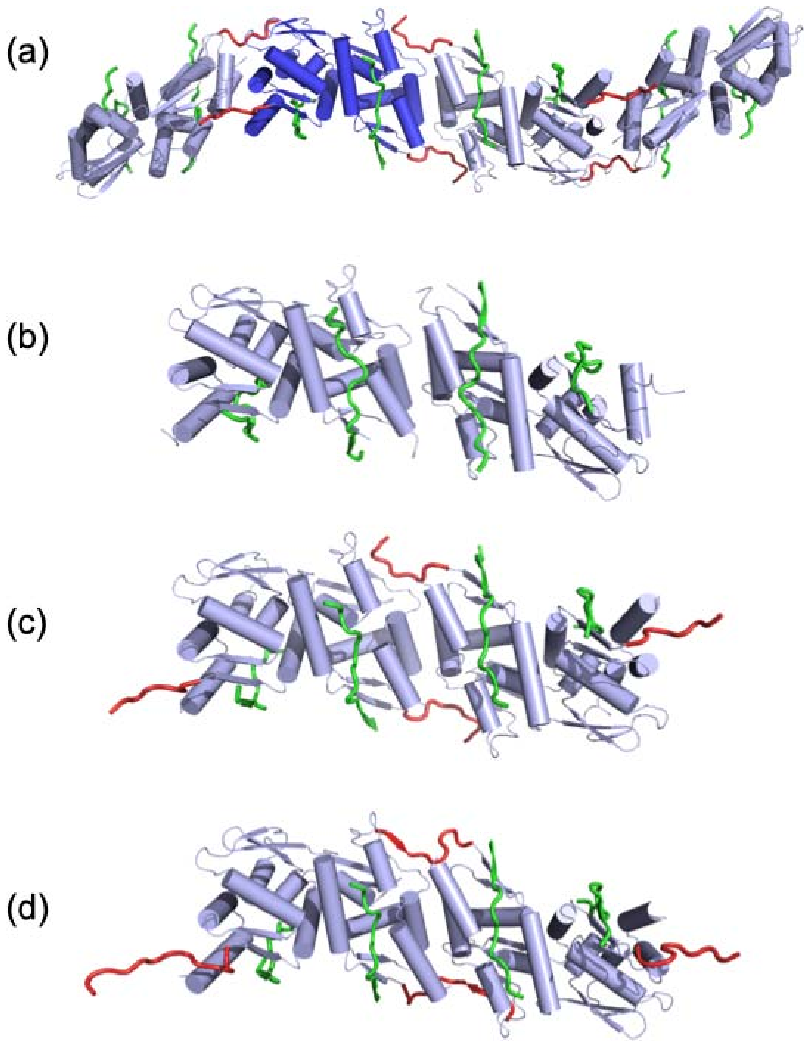

Figure S3 Crystallographic associations between BTB domain dimers. (a) Organization of BTB-domain filaments formed upon crystallization of the NCoR1^{BBD2}-BCL6^{BTB} chimeric protein in the presence of the high-affinity NCoR1^{BBD1} peptide. The filaments comprise four BTB-domain dimers; a single BTB dimer is highlighted in dark blue. (b) Interactions between two BTB-domain dimers in PDB 3BIM; BCOR corepressor residues interact with the lateral groove similarly to the co-repressors SMRT and NCoR1. (c) Interactions between two BTB-domain dimers of the NCoR1^{BBD2}-BCL6^{BTB} chimeric protein crystallized in the presence of the high-affinity NCoR1^{BBD1} peptide. (d) Interactions between two BTB domain dimers of the NCoR1$^{BBD2(link)}$-BCL6^{BTB} chimeric protein crystallized in the presence of the high-affinity NCoR1^{BBD1} peptide. Cartoon representations depict BTB domain sequences in light blue, NCoR1^{BBD2} sequences in red, and NCoR1^{BBD1} (or BCOR) sequences in green. In all cases, the BTB dimers stack together via associations involving the C-terminal α5-α6 helices of adjacent molecules. In (c) and (d), the BBD2 extensions of the chimeric BTB domains interact with the HP regions of adjacent BTB dimers in a process reminiscent of runaway domain coupling; this leads to a stronger interface between units.

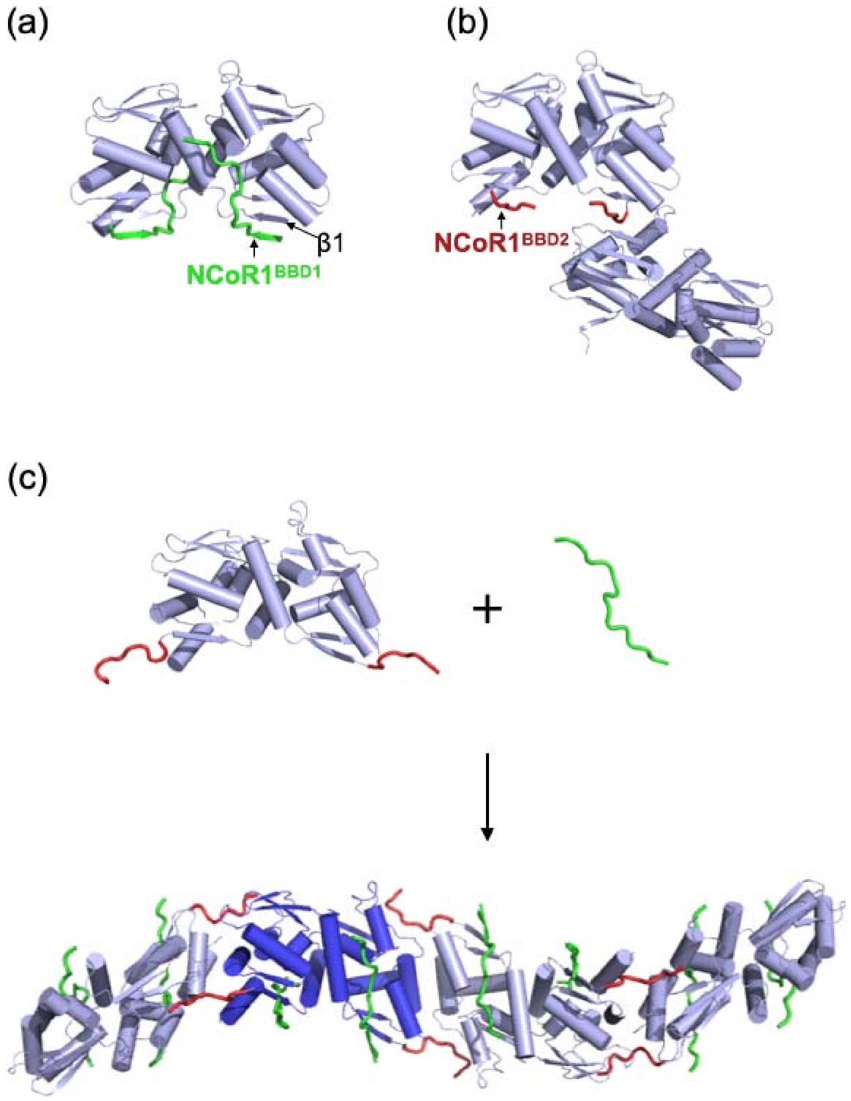

Figure S4 Rationale for the formation of an open crystal lattice containing extended BTB filaments. The high-affinity NCoR1^{BBD1} peptide (green) interacts with the BTB domain lateral groove, which includes the BTB β1-strand. The NCoR1 BBD2 extension (red) of the NCoR1^{BBD2}-BCL6^{BTB} chimeric protein can interact with both the β1-strand and with the hydrophobic patch of the BTB domain, thereby tethering two dimers together in the crystal lattice. When the NCoR1^{BBD2}-BCL6^{BTB} chimeric protein is crystallized in the presence of the high-affinity NCoR1^{BBD1} peptide, the BBD2 extension is precluded from interaction with the BTB β1-strand, and BTB dimers are tethered into filaments via association of their C-terminal α5-α6 helices and via an interaction between the BBD2 extension and the hydrophobic patch.

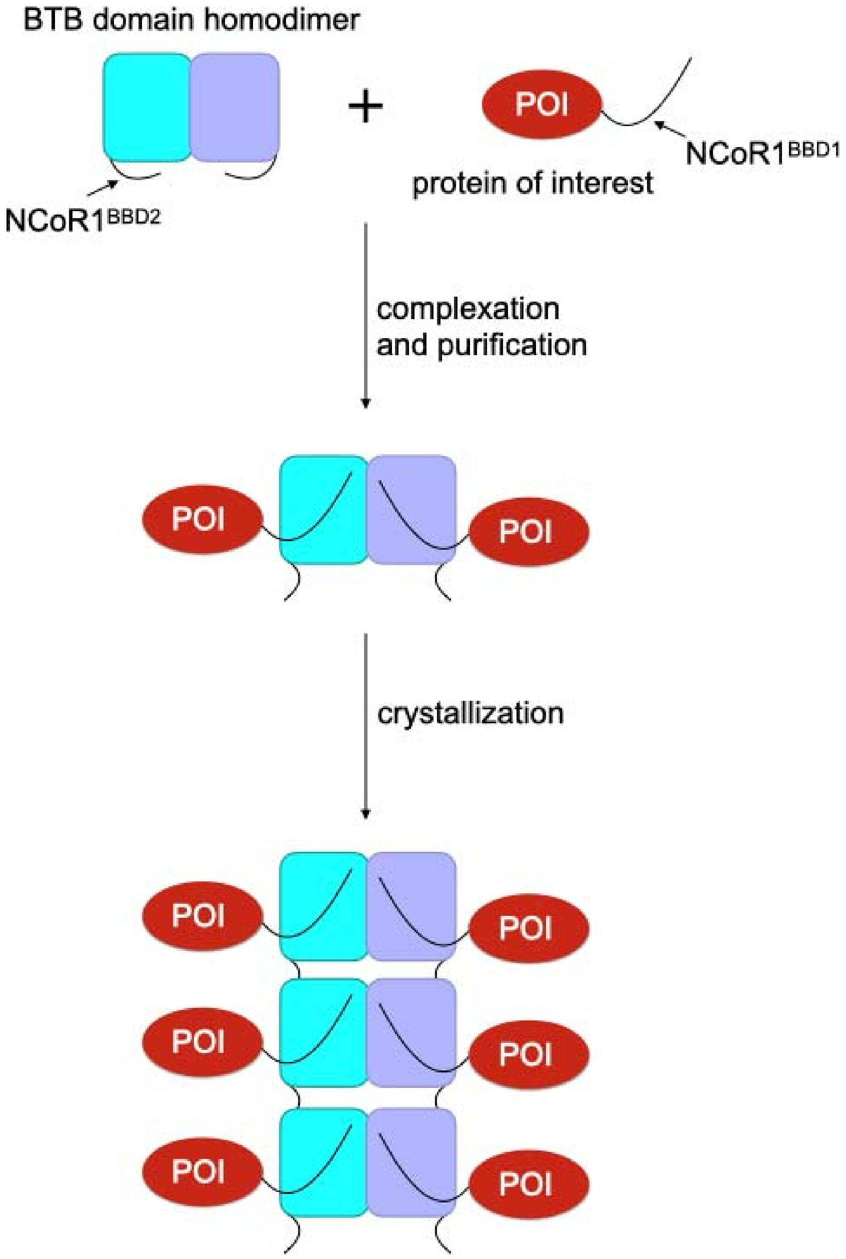

Figure S5 Rationale for using the BTB domain as a crystallization chaperone. The BCL6 BTB domain is expressed with an NCoR1^{BBD2} extension at its N-terminus, and the protein of interest has an NCoR1^{BBD1} extension at its C-terminus. Following co-purification and complexation, the protein of interest is recruited to the BTB domain via its NCoR1^{BBD1} extension. The interactions of the NCoR1^{BBD2} extension facilitate formation of the BTB domain crystal lattice.

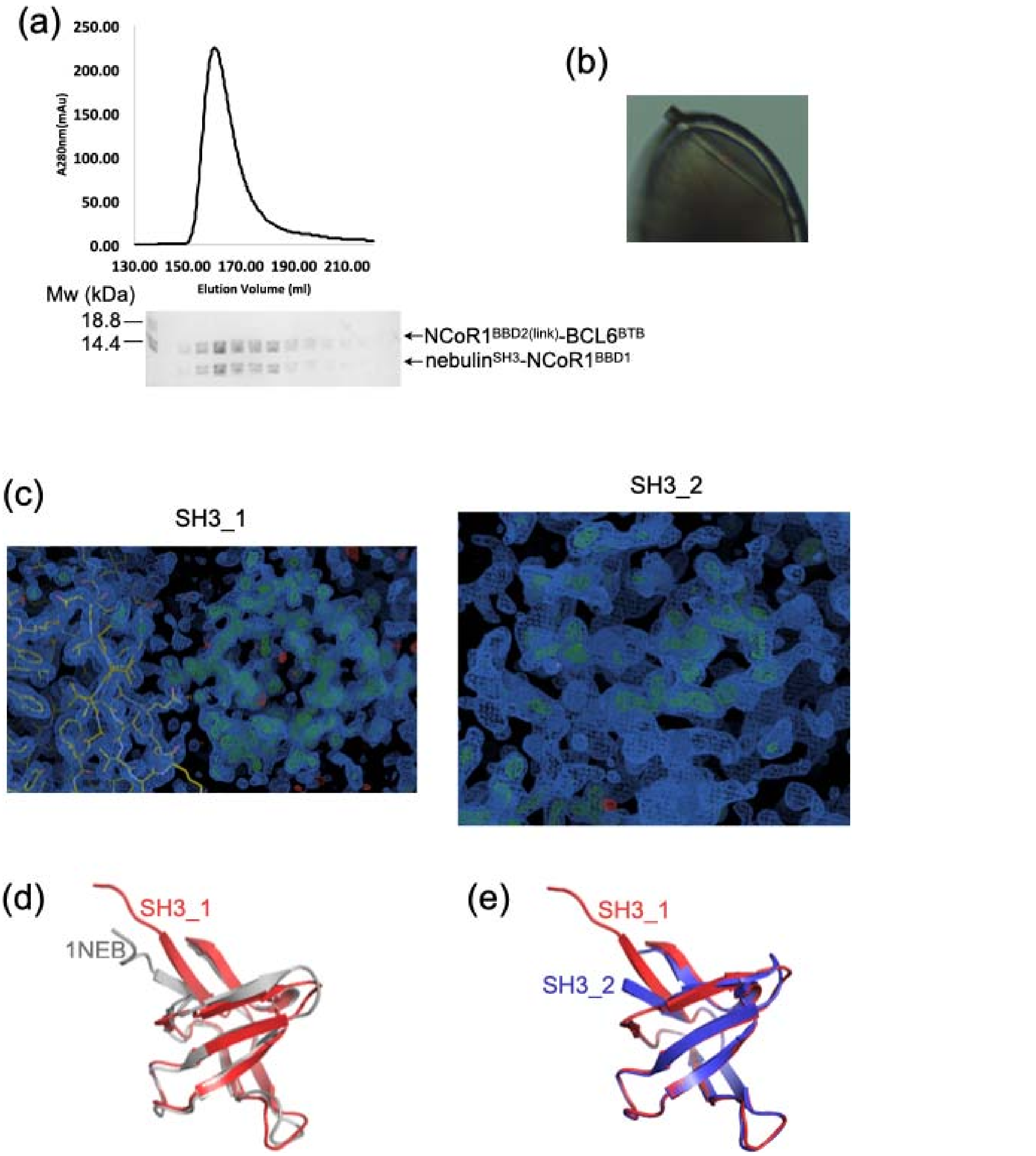

Figure S6 The BCL6 BTB domain as a crystallization chaperone. (a) Co-purification of the NCoR1$^{BBD2(link)}$-BCL6^{BTB} and nebulin^{SH3}-NCoR1^{BBD1} proteins by size-exclusion chromatography. (b) Crystal of the NCoR1$^{BBD2(link)}$-BCL6^{BTB} /nebulin^{SH3}-NCoR1^{BBD1} complex. (c) COOT images of both SH3 domains in the unit cell following a single round of refinement after placement of the BCL6 BTB domain homodimer. The $2F_0$-F_C map is shown in blue and contoured to 1σ, and the F_0-F_C map is contoured to 3.5σ and shown in green. Electron density corresponding to both SH3 domains is clearly visible. (d) Structural superposition of the nebulin SH3_1 domain (red) with the NMR structure (grey; PDB 1NEB). (e) Structural superposition of the two nebulin SH3 domains (SH3_1 and SH3_2) in the unit cell.
